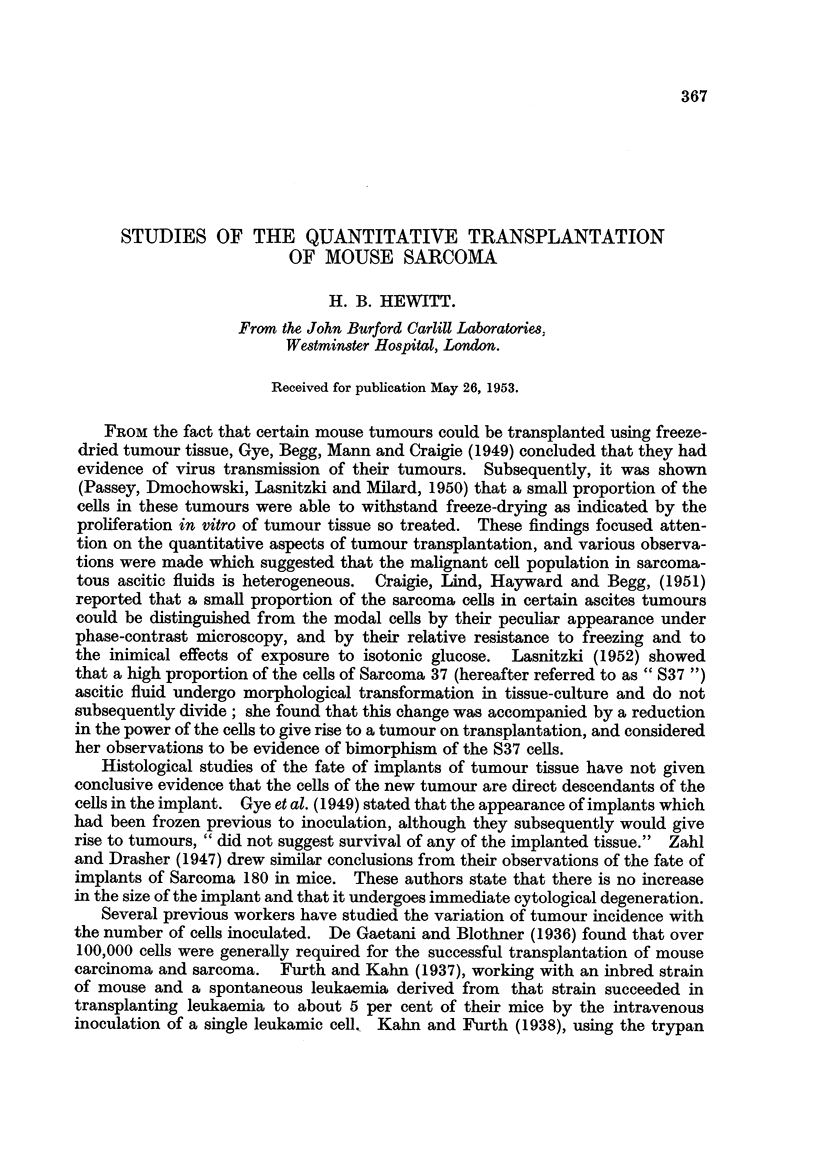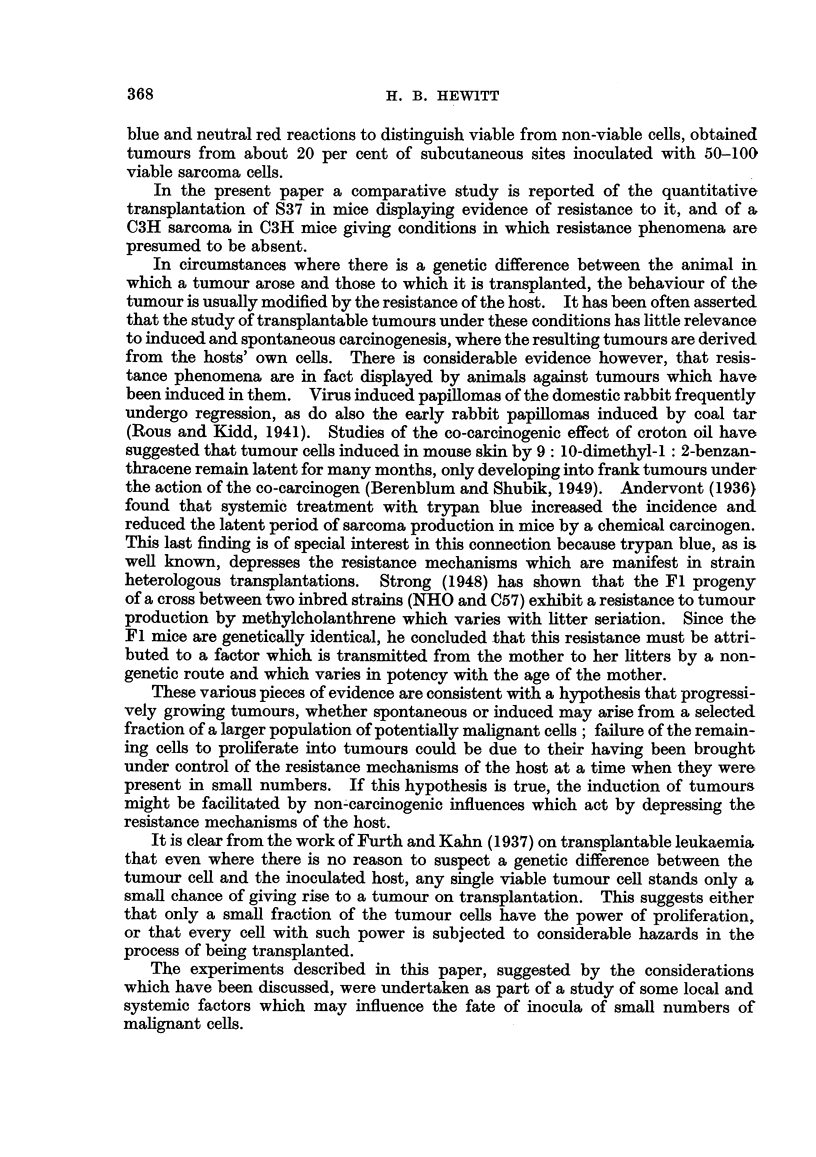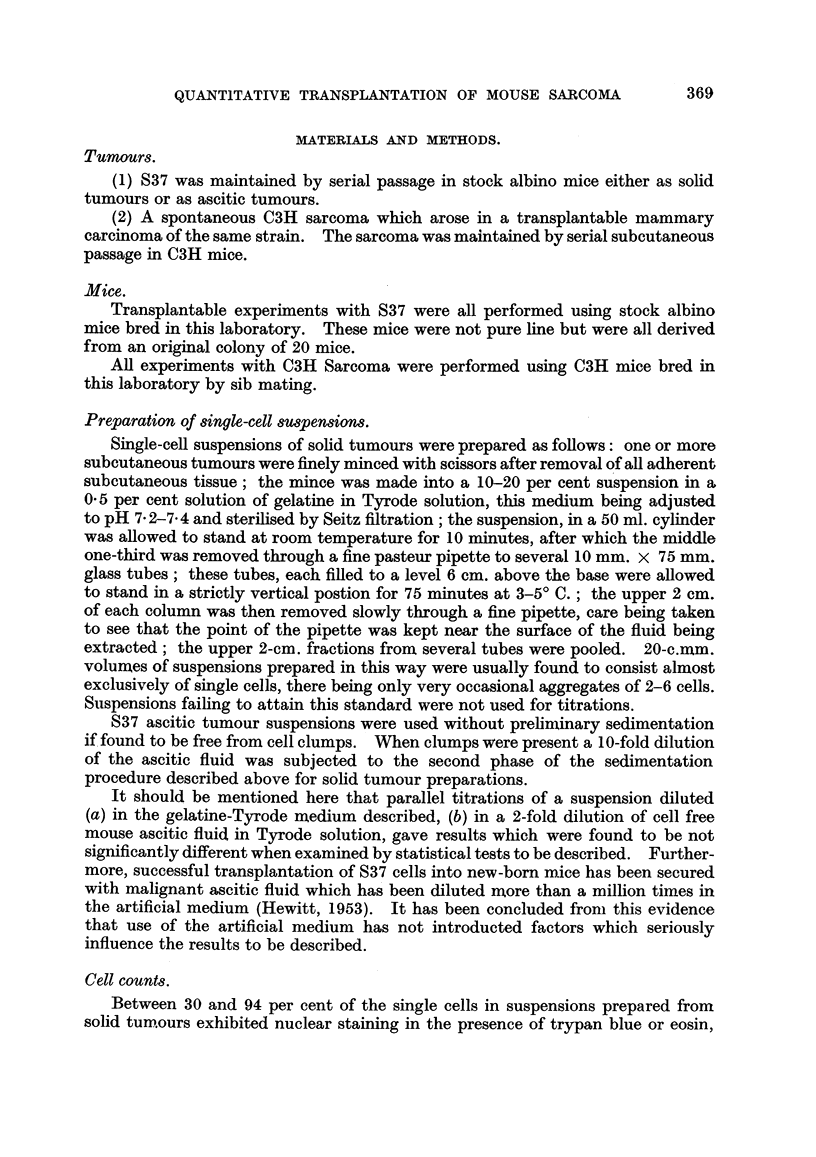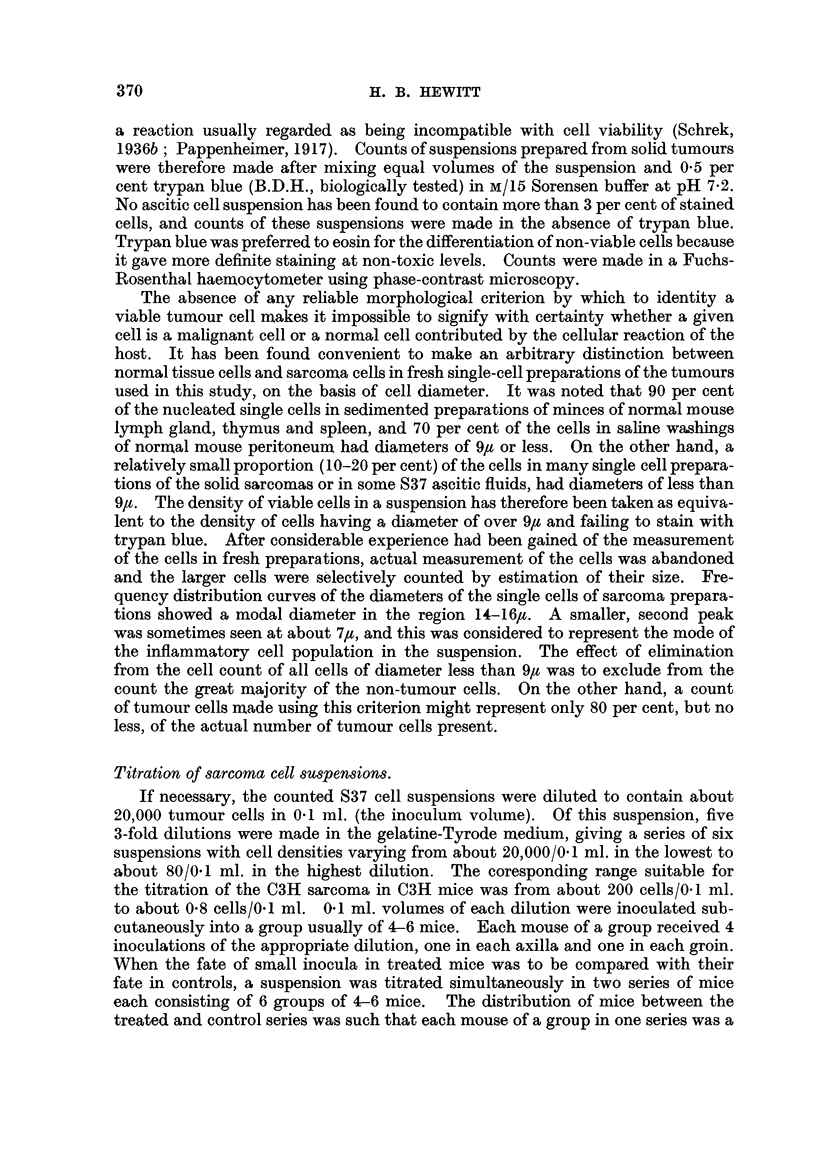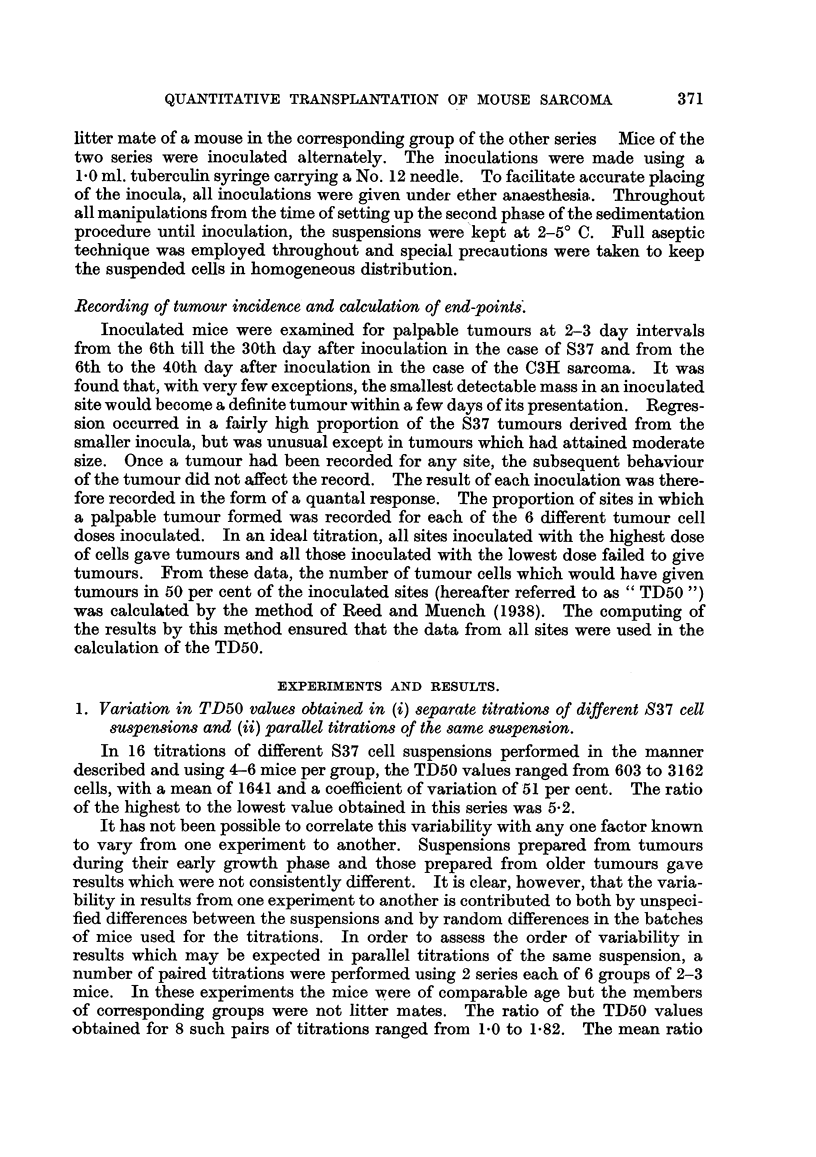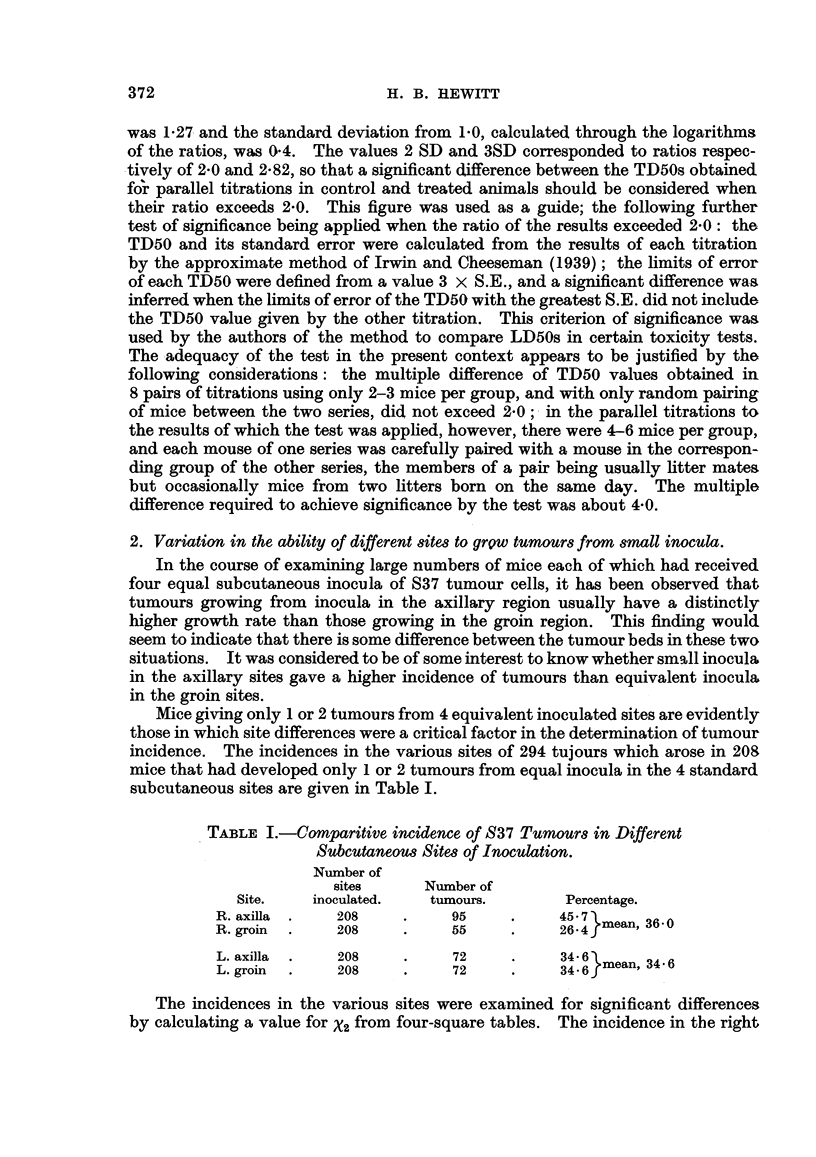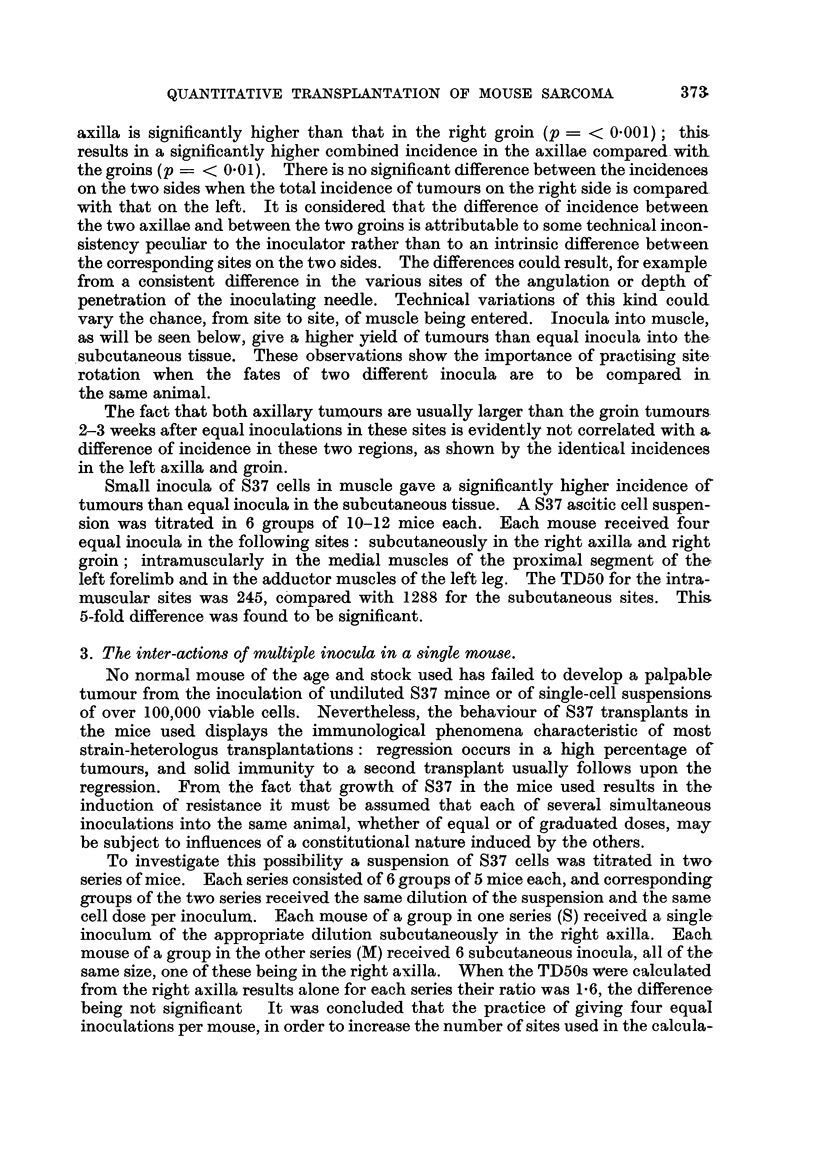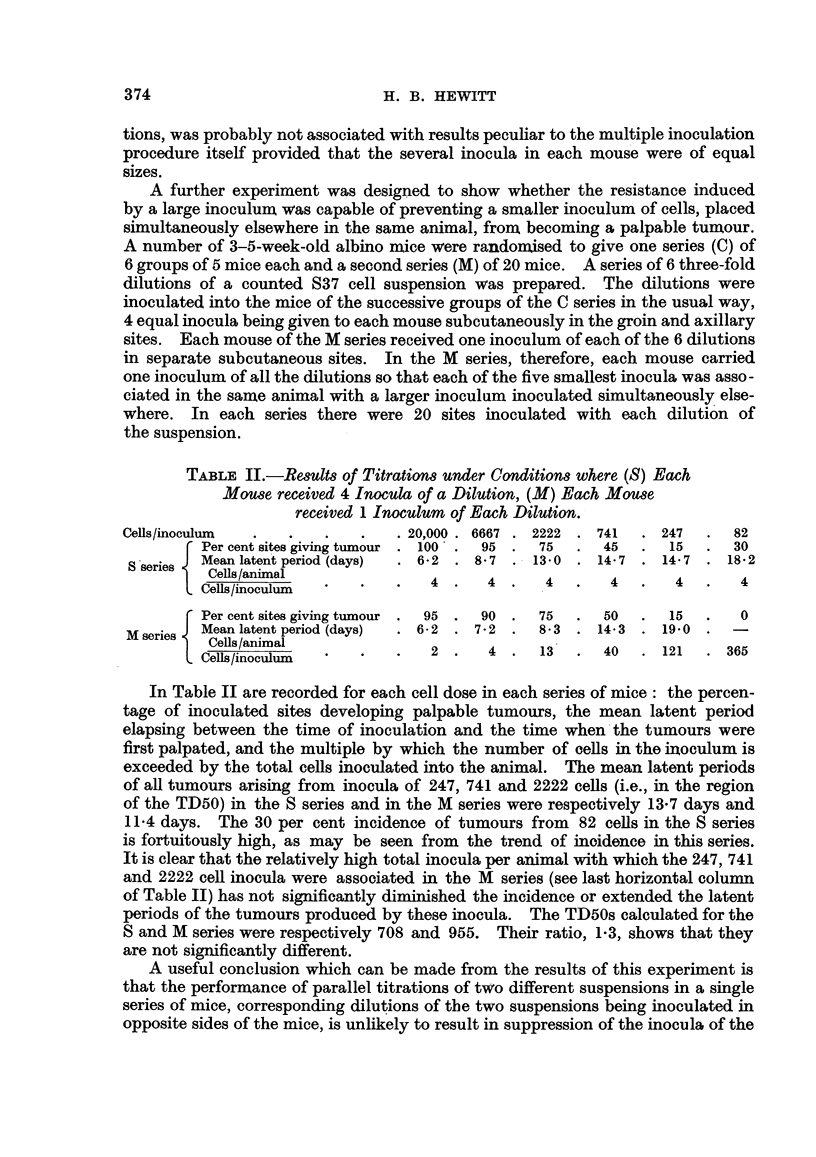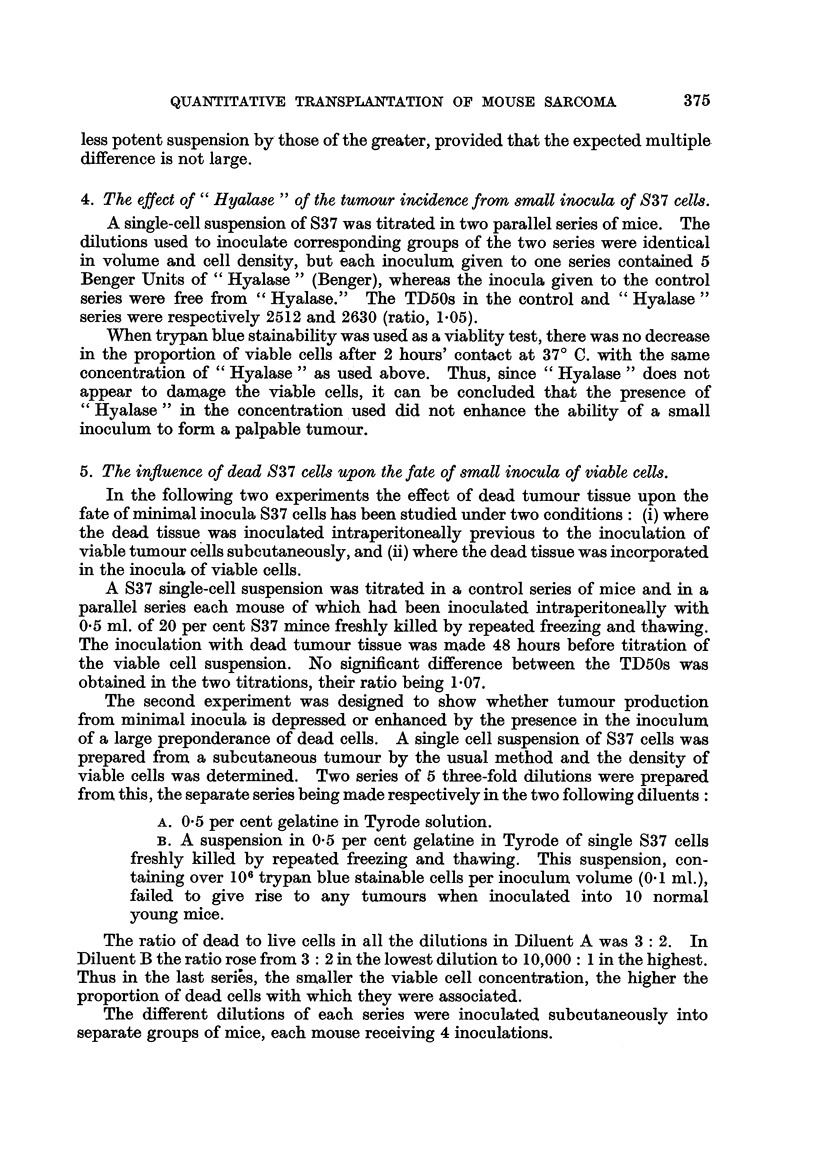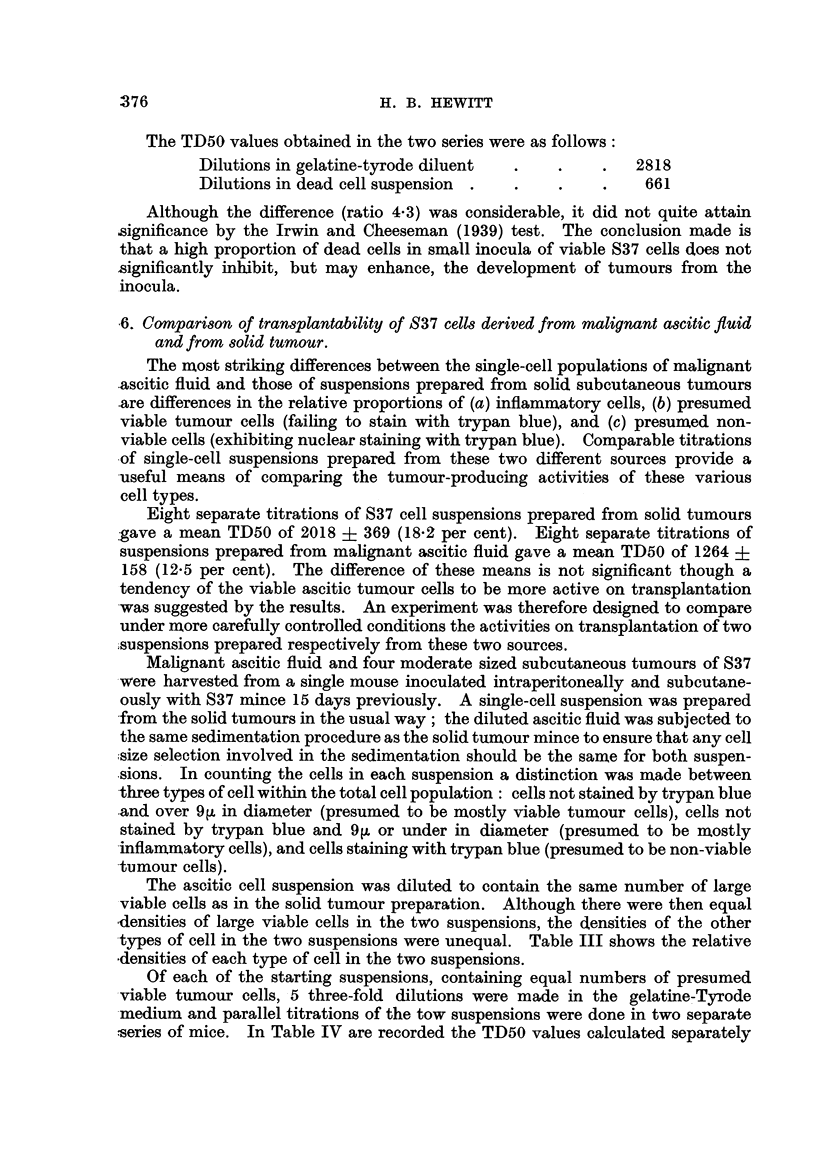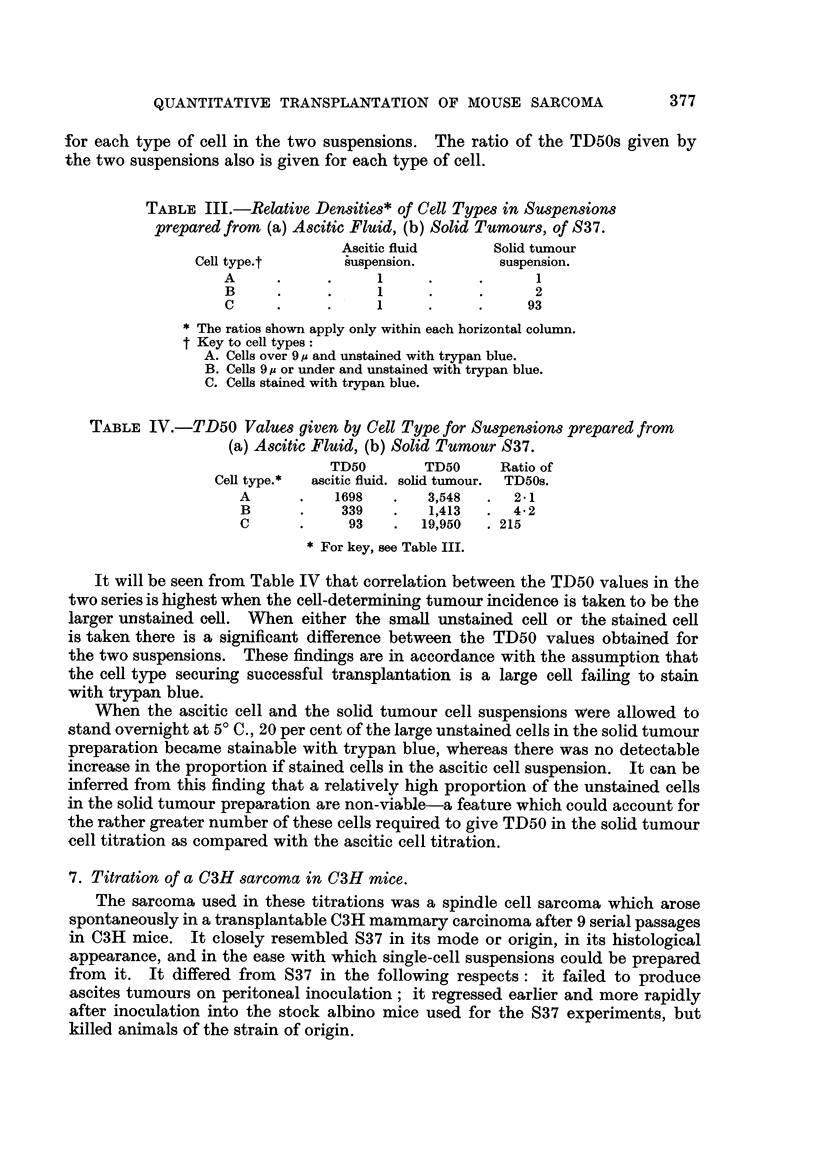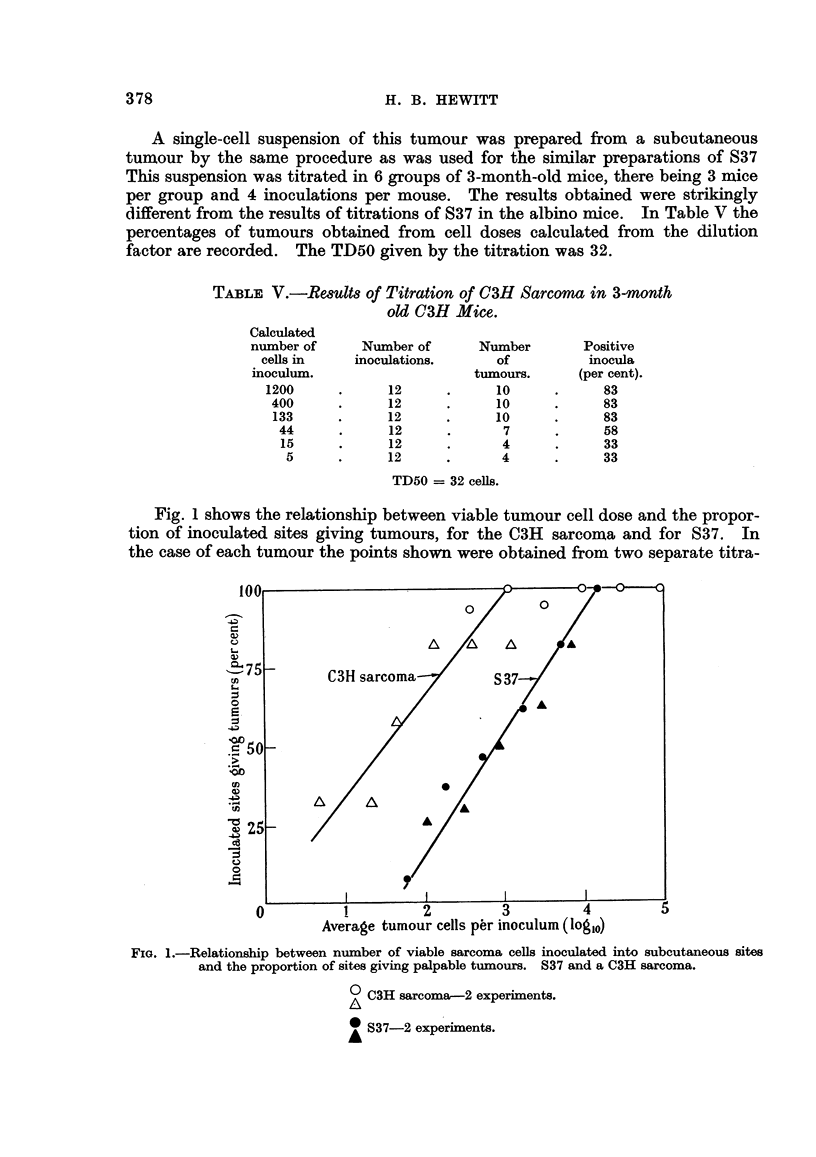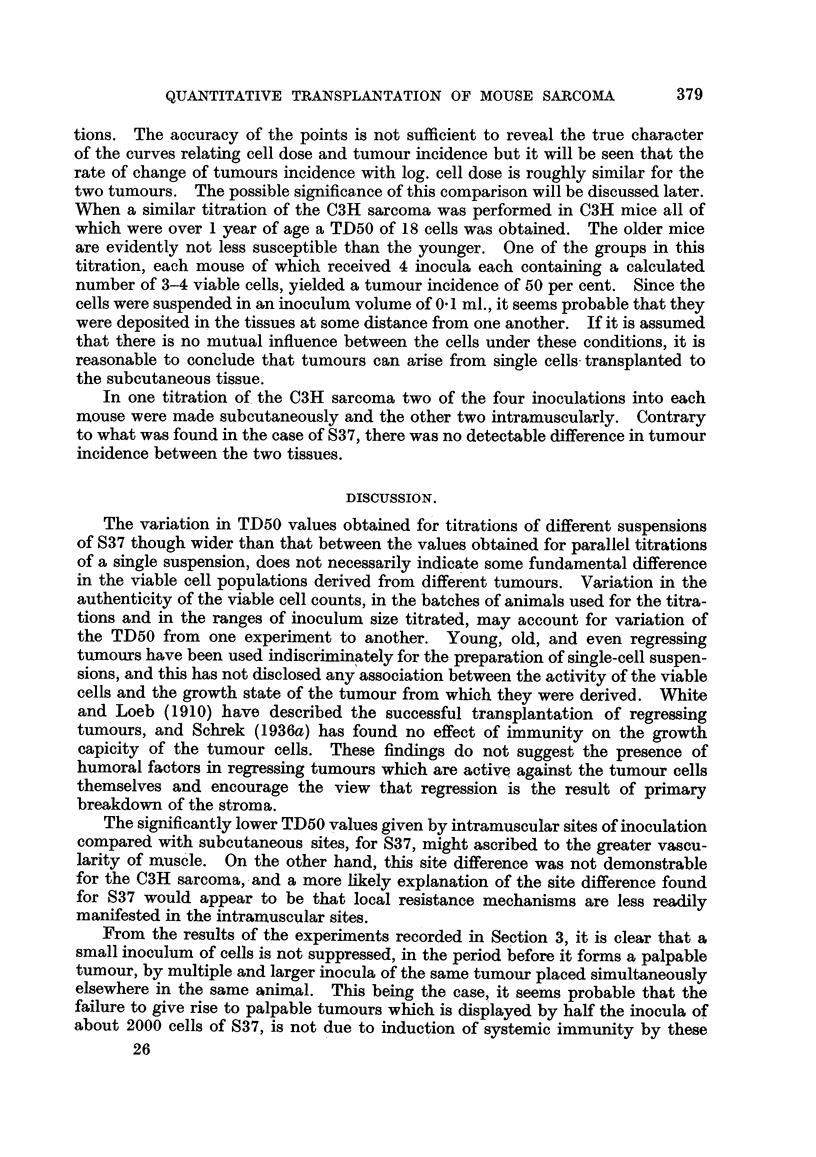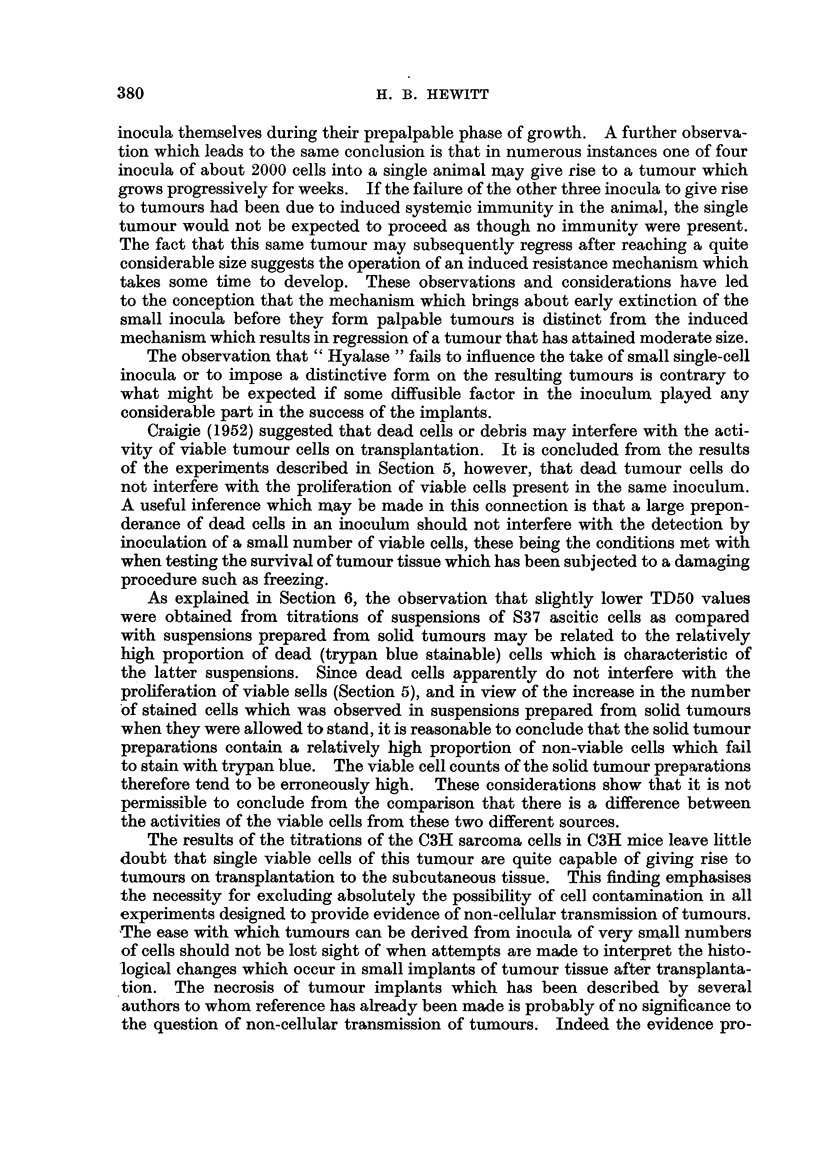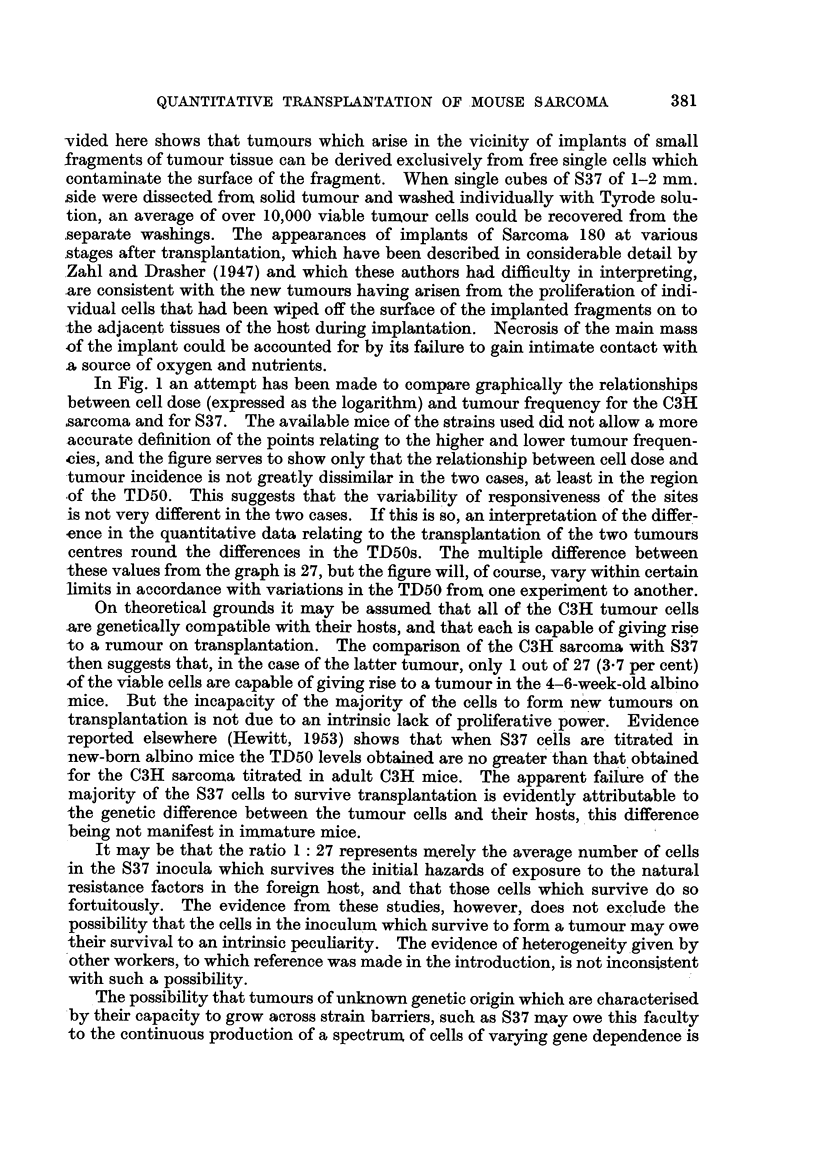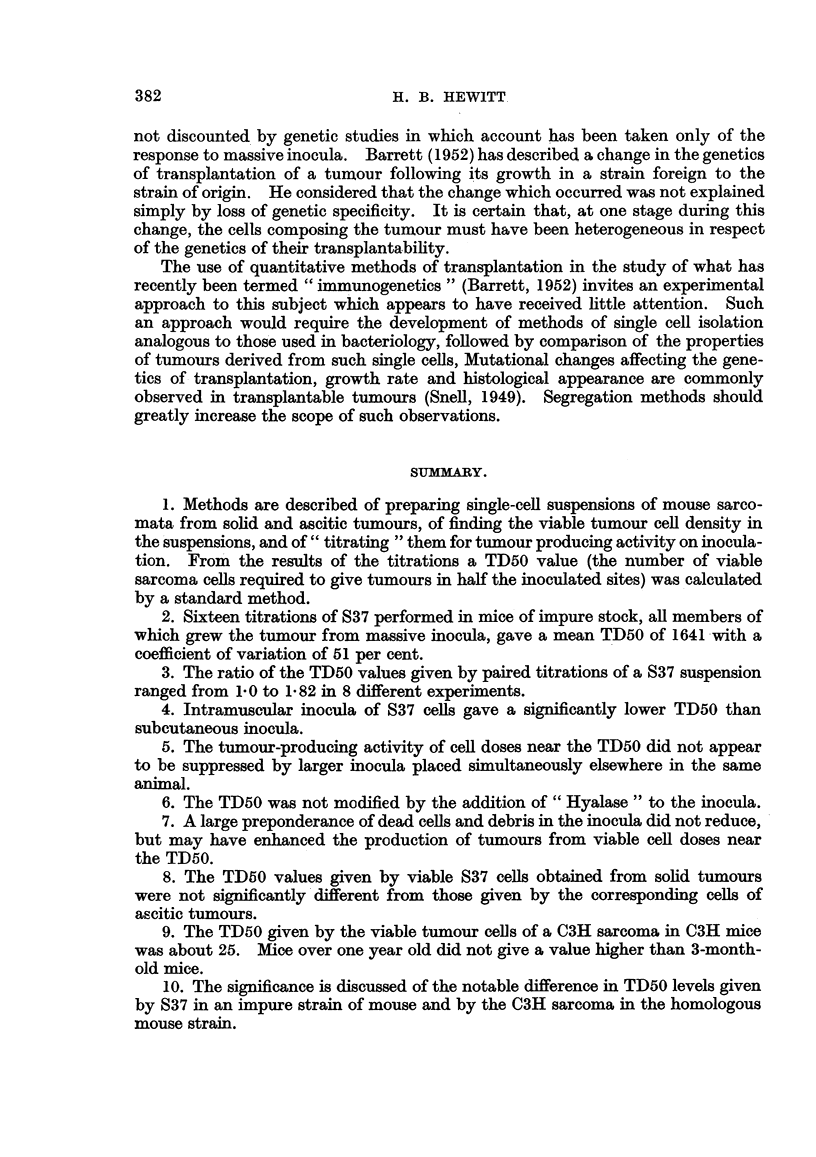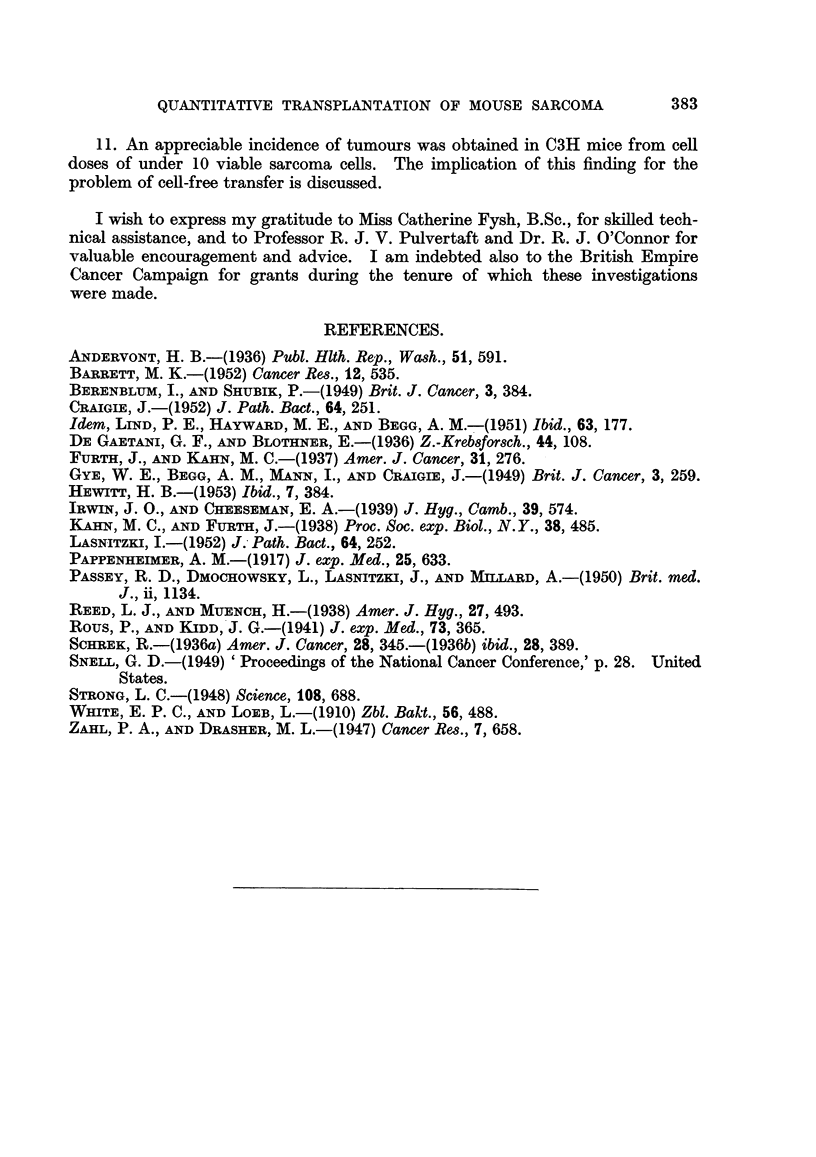# Studies of the Quantitative Transplantation of Mouse Sarcoma

**DOI:** 10.1038/bjc.1953.35

**Published:** 1953-09

**Authors:** H. B. Hewitt


### STUDIES OF THE QUANTITATIVE TRANSPLANTATION OF MOUSE SARCOMA

H. B. HEWITT.

*From the John Burford Carlill Laboratories, Westminster Hospital, London.*

Received for publication May 26, 1953.

FROM the fact that certain mouse tumours could be transplanted using freeze-dried tumour tissue, Gye, Begg, Mann and Craigie (1949) concluded that they had evidence of virus transmission of their tumours. Subsequently, it was shown (Passey, Dmochowski, Lasnitzki and Milard, 1950) that a small proportion of the cells in these tumours were able to withstand freeze-drying as indicated by the proliferation *in vitro* of tumour tissue so treated. These findings focused attention on the quantitative aspects of tumour transplantation, and various observations were made which suggested that the malignant cell population in sarcomatous ascitic fluids is heterogeneous. Craigie, Lind, Hayward and Begg, (1951) reported that a small proportion of the sarcoma cells in certain ascites tumours could be distinguished from the modal cells by their peculiar appearance under phase-contrast microscopy, and by their relative resistance to freezing and to the inimical effects of exposure to isotonic glucose. Lasnitzki (1952) showed that a high proportion of the cells of Sarcoma 37 (hereafter referred to as " S37 ") ascitic fluid undergo morphological transformation in tissue-culture and do not subsequently divide ; she found that this change was accompanied by a reduction in the power of the cells to give rise to a tumour on transplantation, and considered her observations to be evidence of bimorphism of the S37 cells.

Histological studies of the fate of implants of tumour tissue have not given conclusive evidence that the cells of the new tumour are direct descendants of the cells in the implant. Gye *et al.* (1949) stated that the appearance of implants which had been frozen previous to inoculation, although they subsequently would give rise to tumours, " did not suggest survival of any of the implanted tissue." Zahl and Drasher (1947) drew similar conclusions from their observations of the fate of implants of Sarcoma 180 in mice. These authors state that there is no increase in the size of the implant and that it undergoes immediate cytological degeneration.

Several previous workers have studied the variation of tumour incidence with the number of cells inoculated. De Gaetani and Blothner (1936) found that over 100,000 cells were generally required for the successful transplantation of mouse carcinoma and sarcoma. Furth and Kahn (1937), working with an inbred strain of mouse and a spontaneous leukaemia derived from that strain succeeded in transplanting leukaemia to about 5 per cent of their mice by the intravenous inoculation of a single leukamic cell. Kahn and Furth (1938), using the trypan blue and neutral red reactions to distinguish viable from non-viable cells, obtained tumours from about 20 per cent of subcutaneous sites inoculated with 50–100 viable sarcoma cells.

In the present paper a comparative study is reported of the quantitative transplantation of S37 in mice displaying evidence of resistance to it, and of a C3H sarcoma in C3H mice giving conditions in which resistance phenomena are presumed to be absent.

In circumstances where there is a genetic difference between the animal in which a tumour arose and those to which it is transplanted, the behaviour of the tumour is usually modified by the resistance of the host. It has been often asserted that the study of transplantable tumours under these conditions has little relevance to induced and spontaneous carcinogenesis, where the resulting tumours are derived from the hosts' own cells. There is considerable evidence however, that resistance phenomena are in fact displayed by animals against tumours which have been induced in them. Virus induced papillomas of the domestic rabbit frequently undergo regression, as do also the early rabbit papillomas induced by coal tar (Rous and Kidd, 1941). Studies of the co-carcinogenic effect of croton oil have suggested that tumour cells induced in mouse skin by 9 : 10-dimethyl-1 : 2-benzanthracene remain latent for many months, only developing into frank tumours under the action of the co-carcinogen (Berenblum and Shubik, 1949). Andervont (1936) found that systemic treatment with trypan blue increased the incidence and reduced the latent period of sarcoma production in mice by a chemical carcinogen. This last finding is of special interest in this connection because trypan blue, as is well known, depresses the resistance mechanisms which are manifest in strain heterologous transplantations. Strong (1948) has shown that the F1 progeny of a cross between two inbred strains (NHO and C57) exhibit a resistance to tumour production by methylcholanthrene which varies with litter seriation. Since the F1 mice are genetically identical, he concluded that this resistance must be attributed to a factor which is transmitted from the mother to her litters by a non-genetic route and which varies in potency with the age of the mother.

These various pieces of evidence are consistent with a hypothesis that progressively growing tumours, whether spontaneous or induced may arise from a selected fraction of a larger population of potentially malignant cells ; failure of the remaining cells to proliferate into tumours could be due to their having been brought under control of the resistance mechanisms of the host at a time when they were present in small numbers. If this hypothesis is true, the induction of tumours might be facilitated by non-carcinogenic influences which act by depressing the resistance mechanisms of the host.

It is clear from the work of Furth and Kahn (1937) on transplantable leukaemia that even where there is no reason to suspect a genetic difference between the tumour cell and the inoculated host, any single viable tumour cell stands only a small chance of giving rise to a tumour on transplantation. This suggests either that only a small fraction of the tumour cells have the power of proliferation, or that every cell with such power is subjected to considerable hazards in the process of being transplanted.

The experiments described in this paper, suggested by the considerations which have been discussed, were undertaken as part of a study of some local and systemic factors which may influence the fate of inocula of small numbers of malignant cells.

### MATERIALS AND METHODS.

*Tumours.*

(1) S37 was maintained by serial passage in stock albino mice either as solid tumours or as ascitic tumours.

(2) A spontaneous C3H sarcoma which arose in a transplantable mammary carcinoma of the same strain. The sarcoma was maintained by serial subcutaneous passage in C3H mice.

*Mice.*

Transplantable experiments with S37 were all performed using stock albino mice bred in this laboratory. These mice were not pure line but were all derived from an original colony of 20 mice.

All experiments with C3H Sarcoma were performed using C3H mice bred in this laboratory by sib mating.

*Preparation of single-cell suspensions.*

Single-cell suspensions of solid tumours were prepared as follows: one or more subcutaneous tumours were finely minced with scissors after removal of all adherent subcutaneous tissue; the mince was made into a 10–20 per cent suspension in a 0·5 per cent solution of gelatine in Tyrode solution, this medium being adjusted to pH 7·2–7·4 and sterilised by Seitz filtration; the suspension, in a 50 ml. cylinder was allowed to stand at room temperature for 10 minutes, after which the middle one-third was removed through a fine pasteur pipette to several 10 mm. × 75 mm. glass tubes; these tubes, each filled to a level 6 cm. above the base were allowed to stand in a strictly vertical postion for 75 minutes at 3–5° C.; the upper 2 cm. of each column was then removed slowly through a fine pipette, care being taken to see that the point of the pipette was kept near the surface of the fluid being extracted; the upper 2-cm. fractions from several tubes were pooled. 20-c.mm. volumes of suspensions prepared in this way were usually found to consist almost exclusively of single cells, there being only very occasional aggregates of 2–6 cells. Suspensions failing to attain this standard were not used for titrations.

S37 ascitic tumour suspensions were used without preliminary sedimentation if found to be free from cell clumps. When clumps were present a 10-fold dilution of the ascitic fluid was subjected to the second phase of the sedimentation procedure described above for solid tumour preparations.

It should be mentioned here that parallel titrations of a suspension diluted (*a*) in the gelatine-Tyrode medium described, (*b*) in a 2-fold dilution of cell free mouse ascitic fluid in Tyrode solution, gave results which were found to be not significantly different when examined by statistical tests to be described. Furthermore, successful transplantation of S37 cells into new-born mice has been secured with malignant ascitic fluid which has been diluted more than a million times in the artificial medium (Hewitt, 1953). It has been concluded from this evidence that use of the artificial medium has not introducted factors which seriously influence the results to be described.

*Cell counts.*

Between 30 and 94 per cent of the single cells in suspensions prepared from solid tumours exhibited nuclear staining in the presence of trypan blue or eosin, a reaction usually regarded as being incompatible with cell viability (Schrek, 1936*b* ; Pappenheimer, 1917). Counts of suspensions prepared from solid tumours were therefore made after mixing equal volumes of the suspension and 0·5 per cent trypan blue (B.D.H., biologically tested) in M/15 Sorensen buffer at pH 7·2. No ascitic cell suspension has been found to contain more than 3 per cent of stained cells, and counts of these suspensions were made in the absence of trypan blue. Trypan blue was preferred to eosin for the differentiation of non-viable cells because it gave more definite staining at non-toxic levels. Counts were made in a Fuchs-Rosenthal haemocytometer using phase-contrast microscopy.

The absence of any reliable morphological criterion by which to identity a viable tumour cell makes it impossible to signify with certainty whether a given cell is a malignant cell or a normal cell contributed by the cellular reaction of the host. It has been found convenient to make an arbitrary distinction between normal tissue cells and sarcoma cells in fresh single-cell preparations of the tumours used in this study, on the basis of cell diameter. It was noted that 90 per cent of the nucleated single cells in sedimented preparations of minces of normal mouse lymph gland, thymus and spleen, and 70 per cent of the cells in saline washings of normal mouse peritoneum had diameters of $9\mu$ or less. On the other hand, a relatively small proportion (10–20 per cent) of the cells in many single cell preparations of the solid sarcomas or in some S37 ascitic fluids, had diameters of less than $9\mu$. The density of viable cells in a suspension has therefore been taken as equivalent to the density of cells having a diameter of over $9\mu$ and failing to stain with trypan blue. After considerable experience had been gained of the measurement of the cells in fresh preparations, actual measurement of the cells was abandoned and the larger cells were selectively counted by estimation of their size. Frequency distribution curves of the diameters of the single cells of sarcoma preparations showed a modal diameter in the region $14$–$16\mu$. A smaller, second peak was sometimes seen at about $7\mu$, and this was considered to represent the mode of the inflammatory cell population in the suspension. The effect of elimination from the cell count of all cells of diameter less than $9\mu$ was to exclude from the count the great majority of the non-tumour cells. On the other hand, a count of tumour cells made using this criterion might represent only 80 per cent, but no less, of the actual number of tumour cells present.

*Titration of sarcoma cell suspensions.*

If necessary, the counted S37 cell suspensions were diluted to contain about 20,000 tumour cells in 0·1 ml. (the inoculum volume). Of this suspension, five 3-fold dilutions were made in the gelatine-Tyrode medium, giving a series of six suspensions with cell densities varying from about 20,000/0·1 ml. in the lowest to about 80/0·1 ml. in the highest dilution. The coresponding range suitable for the titration of the C3H sarcoma in C3H mice was from about 200 cells/0·1 ml. to about 0·8 cells/0·1 ml. 0·1 ml. volumes of each dilution were inoculated subcutaneously into a group usually of 4–6 mice. Each mouse of a group received 4 inoculations of the appropriate dilution, one in each axilla and one in each groin. When the fate of small inocula in treated mice was to be compared with their fate in controls, a suspension was titrated simultaneously in two series of mice each consisting of 6 groups of 4–6 mice. The distribution of mice between the treated and control series was such that each mouse of a group in one series was a litter mate of a mouse in the corresponding group of the other series Mice of the two series were inoculated alternately. The inoculations were made using a 1·0 ml. tuberculin syringe carrying a No. 12 needle. To facilitate accurate placing of the inocula, all inoculations were given under ether anaesthesia. Throughout all manipulations from the time of setting up the second phase of the sedimentation procedure until inoculation, the suspensions were kept at 2–5° C. Full aseptic technique was employed throughout and special precautions were taken to keep the suspended cells in homogeneous distribution.

*Recording of tumour incidence and calculation of end-points.*

Inoculated mice were examined for palpable tumours at 2–3 day intervals from the 6th till the 30th day after inoculation in the case of S37 and from the 6th to the 40th day after inoculation in the case of the C3H sarcoma. It was found that, with very few exceptions, the smallest detectable mass in an inoculated site would become a definite tumour within a few days of its presentation. Regression occurred in a fairly high proportion of the S37 tumours derived from the smaller inocula, but was unusual except in tumours which had attained moderate size. Once a tumour had been recorded for any site, the subsequent behaviour of the tumour did not affect the record. The result of each inoculation was therefore recorded in the form of a quantal response. The proportion of sites in which a palpable tumour formed was recorded for each of the 6 different tumour cell doses inoculated. In an ideal titration, all sites inoculated with the highest dose of cells gave tumours and all those inoculated with the lowest dose failed to give tumours. From these data, the number of tumour cells which would have given tumours in 50 per cent of the inoculated sites (hereafter referred to as " TD50 ") was calculated by the method of Reed and Muench (1938). The computing of the results by this method ensured that the data from all sites were used in the calculation of the TD50.

### EXPERIMENTS AND RESULTS.

1. *Variation in TD50 values obtained in (i) separate titrations of different S37 cell suspensions and (ii) parallel titrations of the same suspension.*

In 16 titrations of different S37 cell suspensions performed in the manner described and using 4–6 mice per group, the TD50 values ranged from 603 to 3162 cells, with a mean of 1641 and a coefficient of variation of 51 per cent. The ratio of the highest to the lowest value obtained in this series was 5·2.

It has not been possible to correlate this variability with any one factor known to vary from one experiment to another. Suspensions prepared from tumours during their early growth phase and those prepared from older tumours gave results which were not consistently different. It is clear, however, that the variability in results from one experiment to another is contributed to both by unspecified differences between the suspensions and by random differences in the batches of mice used for the titrations. In order to assess the order of variability in results which may be expected in parallel titrations of the same suspension, a number of paired titrations were performed using 2 series each of 6 groups of 2–3 mice. In these experiments the mice were of comparable age but the members of corresponding groups were not litter mates. The ratio of the TD50 values obtained for 8 such pairs of titrations ranged from 1·0 to 1·82. The mean ratio was 1·27 and the standard deviation from 1·0, calculated through the logarithms of the ratios, was 0·4. The values 2 SD and 3SD corresponded to ratios respectively of 2·0 and 2·82, so that a significant difference between the TD50s obtained for parallel titrations in control and treated animals should be considered when their ratio exceeds 2·0. This figure was used as a guide; the following further test of significance being applied when the ratio of the results exceeded 2·0 : the TD50 and its standard error were calculated from the results of each titration by the approximate method of Irwin and Cheeseman (1939) ; the limits of error of each TD50 were defined from a value 3 × S.E., and a significant difference was inferred when the limits of error of the TD50 with the greatest S.E. did not include the TD50 value given by the other titration. This criterion of significance was used by the authors of the method to compare LD50s in certain toxicity tests. The adequacy of the test in the present context appears to be justified by the following considerations : the multiple difference of TD50 values obtained in 8 pairs of titrations using only 2–3 mice per group, and with only random pairing of mice between the two series, did not exceed 2·0 ; in the parallel titrations to the results of which the test was applied, however, there were 4–6 mice per group, and each mouse of one series was carefully paired with a mouse in the corresponding group of the other series, the members of a pair being usually litter mates but occasionally mice from two litters born on the same day. The multiple difference required to achieve significance by the test was about 4·0.

2. *Variation in the ability of different sites to grow tumours from small inocula.*

In the course of examining large numbers of mice each of which had received four equal subcutaneous inocula of S37 tumour cells, it has been observed that tumours growing from inocula in the axillary region usually have a distinctly higher growth rate than those growing in the groin region. This finding would seem to indicate that there is some difference between the tumour beds in these two situations. It was considered to be of some interest to know whether small inocula in the axillary sites gave a higher incidence of tumours than equivalent inocula in the groin sites.

Mice giving only 1 or 2 tumours from 4 equivalent inoculated sites are evidently those in which site differences were a critical factor in the determination of tumour incidence. The incidences in the various sites of 294 tujours which arose in 208 mice that had developed only 1 or 2 tumours from equal inocula in the 4 standard subcutaneous sites are given in Table I.

TABLE I.—*Comparitive incidence of S37 Tumours in Different Subcutaneous Sites of Inoculation.*

| Site. | Number of sites inoculated. | Number of tumours. | Percentage. | |
|---|---|---|---|---|
| R. axilla | 208 | 95 | 45·7 | mean, 36·0 |
| R. groin | 208 | 55 | 26·4 | |
| L. axilla | 208 | 72 | 34·6 | mean, 34·6 |
| L. groin | 208 | 72 | 34·6 | |

The incidences in the various sites were examined for significant differences by calculating a value for $\chi_2$ from four-square tables. The incidence in the right axilla is significantly higher than that in the right groin ($p = < 0 \cdot 001$); this results in a significantly higher combined incidence in the axillae compared with the groins ($p = < 0 \cdot 01$). There is no significant difference between the incidences on the two sides when the total incidence of tumours on the right side is compared with that on the left. It is considered that the difference of incidence between the two axillae and between the two groins is attributable to some technical inconsistency peculiar to the inoculator rather than to an intrinsic difference between the corresponding sites on the two sides. The differences could result, for example from a consistent difference in the various sites of the angulation or depth of penetration of the inoculating needle. Technical variations of this kind could vary the chance, from site to site, of muscle being entered. Inocula into muscle, as will be seen below, give a higher yield of tumours than equal inocula into the subcutaneous tissue. These observations show the importance of practising site rotation when the fates of two different inocula are to be compared in the same animal.

The fact that both axillary tumours are usually larger than the groin tumours 2–3 weeks after equal inoculations in these sites is evidently not correlated with a difference of incidence in these two regions, as shown by the identical incidences in the left axilla and groin.

Small inocula of S37 cells in muscle gave a significantly higher incidence of tumours than equal inocula in the subcutaneous tissue. A S37 ascitic cell suspension was titrated in 6 groups of 10–12 mice each. Each mouse received four equal inocula in the following sites: subcutaneously in the right axilla and right groin; intramuscularly in the medial muscles of the proximal segment of the left forelimb and in the adductor muscles of the left leg. The TD50 for the intramuscular sites was 245, compared with 1288 for the subcutaneous sites. This 5-fold difference was found to be significant.

3. *The inter-actions of multiple inocula in a single mouse.*

No normal mouse of the age and stock used has failed to develop a palpable tumour from the inoculation of undiluted S37 mince or of single-cell suspensions of over 100,000 viable cells. Nevertheless, the behaviour of S37 transplants in the mice used displays the immunological phenomena characteristic of most strain-heterologus transplantations: regression occurs in a high percentage of tumours, and solid immunity to a second transplant usually follows upon the regression. From the fact that growth of S37 in the mice used results in the induction of resistance it must be assumed that each of several simultaneous inoculations into the same animal, whether of equal or of graduated doses, may be subject to influences of a constitutional nature induced by the others.

To investigate this possibility a suspension of S37 cells was titrated in two series of mice. Each series consisted of 6 groups of 5 mice each, and corresponding groups of the two series received the same dilution of the suspension and the same cell dose per inoculum. Each mouse of a group in one series (S) received a single inoculum of the appropriate dilution subcutaneously in the right axilla. Each mouse of a group in the other series (M) received 6 subcutaneous inocula, all of the same size, one of these being in the right axilla. When the TD50s were calculated from the right axilla results alone for each series their ratio was 1·6, the difference being not significant It was concluded that the practice of giving four equal inoculations per mouse, in order to increase the number of sites used in the calculations, was probably not associated with results peculiar to the multiple inoculation procedure itself provided that the several inocula in each mouse were of equal sizes.

A further experiment was designed to show whether the resistance induced by a large inoculum was capable of preventing a smaller inoculum of cells, placed simultaneously elsewhere in the same animal, from becoming a palpable tumour. A number of 3–5-week-old albino mice were randomised to give one series (C) of 6 groups of 5 mice each and a second series (M) of 20 mice. A series of 6 three-fold dilutions of a counted S37 cell suspension was prepared. The dilutions were inoculated into the mice of the successive groups of the C series in the usual way, 4 equal inocula being given to each mouse subcutaneously in the groin and axillary sites. Each mouse of the M series received one inoculum of each of the 6 dilutions in separate subcutaneous sites. In the M series, therefore, each mouse carried one inoculum of all the dilutions so that each of the five smallest inocula was associated in the same animal with a larger inoculum inoculated simultaneously elsewhere. In each series there were 20 sites inoculated with each dilution of the suspension.

TABLE II.—*Results of Titrations under Conditions where (S) Each Mouse received 4 Inocula of a Dilution, (M) Each Mouse received 1 Inoculum of Each Dilution.*

| | | | | | | | |
|---|---|---|---|---|---|---|---|
| Cells/inoculum | | 20,000 | 6667 | 2222 | 741 | 247 | 82 |
| S series | Per cent sites giving tumour | 100 | 95 | 75 | 45 | 15 | 30 |
| | Mean latent period (days) | 6·2 | 8·7 | 13·0 | 14·7 | 14·7 | 18·2 |
| | Cells/animal / Cells/inoculum | 4 | 4 | 4 | 4 | 4 | 4 |
| M series | Per cent sites giving tumour | 95 | 90 | 75 | 50 | 15 | 0 |
| | Mean latent period (days) | 6·2 | 7·2 | 8·3 | 14·3 | 19·0 | — |
| | Cells/animal / Cells/inoculum | 2 | 4 | 13 | 40 | 121 | 365 |

In Table II are recorded for each cell dose in each series of mice : the percentage of inoculated sites developing palpable tumours, the mean latent period elapsing between the time of inoculation and the time when the tumours were first palpated, and the multiple by which the number of cells in the inoculum is exceeded by the total cells inoculated into the animal. The mean latent periods of all tumours arising from inocula of 247, 741 and 2222 cells (i.e., in the region of the TD50) in the S series and in the M series were respectively 13·7 days and 11·4 days. The 30 per cent incidence of tumours from 82 cells in the S series is fortuitously high, as may be seen from the trend of incidence in this series. It is clear that the relatively high total inocula per animal with which the 247, 741 and 2222 cell inocula were associated in the M series (see last horizontal column of Table II) has not significantly diminished the incidence or extended the latent periods of the tumours produced by these inocula. The TD50s calculated for the S and M series were respectively 708 and 955. Their ratio, 1·3, shows that they are not significantly different.

A useful conclusion which can be made from the results of this experiment is that the performance of parallel titrations of two different suspensions in a single series of mice, corresponding dilutions of the two suspensions being inoculated in opposite sides of the mice, is unlikely to result in suppression of the inocula of the less potent suspension by those of the greater, provided that the expected multiple difference is not large.

4. *The effect of " Hyalase " of the tumour incidence from small inocula of S37 cells.*

A single-cell suspension of S37 was titrated in two parallel series of mice. The dilutions used to inoculate corresponding groups of the two series were identical in volume and cell density, but each inoculum given to one series contained 5 Benger Units of " Hyalase " (Benger), whereas the inocula given to the control series were free from " Hyalase." The TD50s in the control and " Hyalase " series were respectively 2512 and 2630 (ratio, 1·05).

When trypan blue stainability was used as a viablity test, there was no decrease in the proportion of viable cells after 2 hours' contact at 37° C. with the same concentration of " Hyalase " as used above. Thus, since " Hyalase " does not appear to damage the viable cells, it can be concluded that the presence of " Hyalase " in the concentration used did not enhance the ability of a small inoculum to form a palpable tumour.

5. *The influence of dead S37 cells upon the fate of small inocula of viable cells.*

In the following two experiments the effect of dead tumour tissue upon the fate of minimal inocula S37 cells has been studied under two conditions : (i) where the dead tissue was inoculated intraperitoneally previous to the inoculation of viable tumour cells subcutaneously, and (ii) where the dead tissue was incorporated in the inocula of viable cells.

A S37 single-cell suspension was titrated in a control series of mice and in a parallel series each mouse of which had been inoculated intraperitoneally with 0·5 ml. of 20 per cent S37 mince freshly killed by repeated freezing and thawing. The inoculation with dead tumour tissue was made 48 hours before titration of the viable cell suspension. No significant difference between the TD50s was obtained in the two titrations, their ratio being 1·07.

The second experiment was designed to show whether tumour production from minimal inocula is depressed or enhanced by the presence in the inoculum of a large preponderance of dead cells. A single cell suspension of S37 cells was prepared from a subcutaneous tumour by the usual method and the density of viable cells was determined. Two series of 5 three-fold dilutions were prepared from this, the separate series being made respectively in the two following diluents :

A. 0·5 per cent gelatine in Tyrode solution.

B. A suspension in 0·5 per cent gelatine in Tyrode of single S37 cells freshly killed by repeated freezing and thawing. This suspension, containing over $10^6$ trypan blue stainable cells per inoculum volume (0·1 ml.), failed to give rise to any tumours when inoculated into 10 normal young mice.

The ratio of dead to live cells in all the dilutions in Diluent A was 3 : 2. In Diluent B the ratio rose from 3 : 2 in the lowest dilution to 10,000 : 1 in the highest. Thus in the last series, the smaller the viable cell concentration, the higher the proportion of dead cells with which they were associated.

The different dilutions of each series were inoculated subcutaneously into separate groups of mice, each mouse receiving 4 inoculations.

The TD50 values obtained in the two series were as follows :

| | |
|---|---|
| Dilutions in gelatine-tyrode diluent . . . . | 2818 |
| Dilutions in dead cell suspension . . . . . | 661 |

Although the difference (ratio 4·3) was considerable, it did not quite attain significance by the Irwin and Cheeseman (1939) test. The conclusion made is that a high proportion of dead cells in small inocula of viable S37 cells does not significantly inhibit, but may enhance, the development of tumours from the inocula.

6. *Comparison of transplantability of S*37 *cells derived from malignant ascitic fluid and from solid tumour.*

The most striking differences between the single-cell populations of malignant ascitic fluid and those of suspensions prepared from solid subcutaneous tumours are differences in the relative proportions of (*a*) inflammatory cells, (*b*) presumed viable tumour cells (failing to stain with trypan blue), and (*c*) presumed non-viable cells (exhibiting nuclear staining with trypan blue). Comparable titrations of single-cell suspensions prepared from these two different sources provide a useful means of comparing the tumour-producing activities of these various cell types.

Eight separate titrations of S37 cell suspensions prepared from solid tumours gave a mean TD50 of 2018 $\pm$ 369 (18·2 per cent). Eight separate titrations of suspensions prepared from malignant ascitic fluid gave a mean TD50 of 1264 $\pm$ 158 (12·5 per cent). The difference of these means is not significant though a tendency of the viable ascitic tumour cells to be more active on transplantation was suggested by the results. An experiment was therefore designed to compare under more carefully controlled conditions the activities on transplantation of two suspensions prepared respectively from these two sources.

Malignant ascitic fluid and four moderate sized subcutaneous tumours of S37 were harvested from a single mouse inoculated intraperitoneally and subcutaneously with S37 mince 15 days previously. A single-cell suspension was prepared from the solid tumours in the usual way ; the diluted ascitic fluid was subjected to the same sedimentation procedure as the solid tumour mince to ensure that any cell size selection involved in the sedimentation should be the same for both suspensions. In counting the cells in each suspension a distinction was made between three types of cell within the total cell population : cells not stained by trypan blue and over 9$\mu$ in diameter (presumed to be mostly viable tumour cells), cells not stained by trypan blue and 9$\mu$ or under in diameter (presumed to be mostly inflammatory cells), and cells staining with trypan blue (presumed to be non-viable tumour cells).

The ascitic cell suspension was diluted to contain the same number of large viable cells as in the solid tumour preparation. Although there were then equal densities of large viable cells in the two suspensions, the densities of the other types of cell in the two suspensions were unequal. Table III shows the relative densities of each type of cell in the two suspensions.

Of each of the starting suspensions, containing equal numbers of presumed viable tumour cells, 5 three-fold dilutions were made in the gelatine-Tyrode medium and parallel titrations of the tow suspensions were done in two separate series of mice. In Table IV are recorded the TD50 values calculated separately for each type of cell in the two suspensions. The ratio of the TD50s given by the two suspensions also is given for each type of cell.

TABLE III.—*Relative Densities* of Cell Types in Suspensions prepared from* (a) *Ascitic Fluid,* (b) *Solid Tumours, of S*37.

| Cell type.† | Ascitic fluid suspension. | Solid tumour suspension. |
|---|---|---|
| A | 1 | 1 |
| B | 1 | 2 |
| C | 1 | 93 |

* The ratios shown apply only within each horizontal column.
† Key to cell types :
A. Cells over 9 μ and unstained with trypan blue.
B. Cells 9 μ or under and unstained with trypan blue.
C. Cells stained with trypan blue.

TABLE IV.—*TD*50 *Values given by Cell Type for Suspensions prepared from* (a) *Ascitic Fluid,* (b) *Solid Tumour S*37.

| Cell type.* | TD50 ascitic fluid. | TD50 solid tumour. | Ratio of TD50s. |
|---|---|---|---|
| A | 1698 | 3,548 | 2·1 |
| B | 339 | 1,413 | 4·2 |
| C | 93 | 19,950 | 215 |

* For key, see Table III.

It will be seen from Table IV that correlation between the TD50 values in the two series is highest when the cell-determining tumour incidence is taken to be the larger unstained cell. When either the small unstained cell or the stained cell is taken there is a significant difference between the TD50 values obtained for the two suspensions. These findings are in accordance with the assumption that the cell type securing successful transplantation is a large cell failing to stain with trypan blue.

When the ascitic cell and the solid tumour cell suspensions were allowed to stand overnight at 5° C., 20 per cent of the large unstained cells in the solid tumour preparation became stainable with trypan blue, whereas there was no detectable increase in the proportion if stained cells in the ascitic cell suspension. It can be inferred from this finding that a relatively high proportion of the unstained cells in the solid tumour preparation are non-viable—a feature which could account for the rather greater number of these cells required to give TD50 in the solid tumour cell titration as compared with the ascitic cell titration.

7. *Titration of a C3H sarcoma in C3H mice.*

The sarcoma used in these titrations was a spindle cell sarcoma which arose spontaneously in a transplantable C3H mammary carcinoma after 9 serial passages in C3H mice. It closely resembled S37 in its mode or origin, in its histological appearance, and in the ease with which single-cell suspensions could be prepared from it. It differed from S37 in the following respects : it failed to produce ascites tumours on peritoneal inoculation ; it regressed earlier and more rapidly after inoculation into the stock albino mice used for the S37 experiments, but killed animals of the strain of origin.

A single-cell suspension of this tumour was prepared from a subcutaneous tumour by the same procedure as was used for the similar preparations of S37 This suspension was titrated in 6 groups of 3-month-old mice, there being 3 mice per group and 4 inoculations per mouse. The results obtained were strikingly different from the results of titrations of S37 in the albino mice. In Table V the percentages of tumours obtained from cell doses calculated from the dilution factor are recorded. The TD50 given by the titration was 32.

TABLE V.—*Results of Titration of C3H Sarcoma in 3-month old C3H Mice.*

| Calculated number of cells in inoculum. | Number of inoculations. | Number of tumours. | Positive inocula (per cent). |
|---|---|---|---|
| 1200 | 12 | 10 | 83 |
| 400 | 12 | 10 | 83 |
| 133 | 12 | 10 | 83 |
| 44 | 12 | 7 | 58 |
| 15 | 12 | 4 | 33 |
| 5 | 12 | 4 | 33 |

TD50 = 32 cells.

Fig. 1 shows the relationship between viable tumour cell dose and the proportion of inoculated sites giving tumours, for the C3H sarcoma and for S37. In the case of each tumour the points shown were obtained from two separate titra-

FIG. 1.—Relationship between number of viable sarcoma cells inoculated into subcutaneous sites and the proportion of sites giving palpable tumours. S37 and a C3H sarcoma.

○ △ C3H sarcoma—2 experiments.

● ▲ S37—2 experiments.

tions. The accuracy of the points is not sufficient to reveal the true character of the curves relating cell dose and tumour incidence but it will be seen that the rate of change of tumours incidence with log. cell dose is roughly similar for the two tumours. The possible significance of this comparison will be discussed later. When a similar titration of the C3H sarcoma was performed in C3H mice all of which were over 1 year of age a TD50 of 18 cells was obtained. The older mice are evidently not less susceptible than the younger. One of the groups in this titration, each mouse of which received 4 inocula each containing a calculated number of 3–4 viable cells, yielded a tumour incidence of 50 per cent. Since the cells were suspended in an inoculum volume of 0·1 ml., it seems probable that they were deposited in the tissues at some distance from one another. If it is assumed that there is no mutual influence between the cells under these conditions, it is reasonable to conclude that tumours can arise from single cells transplanted to the subcutaneous tissue.

In one titration of the C3H sarcoma two of the four inoculations into each mouse were made subcutaneously and the other two intramuscularly. Contrary to what was found in the case of S37, there was no detectable difference in tumour incidence between the two tissues.

### DISCUSSION.

The variation in TD50 values obtained for titrations of different suspensions of S37 though wider than that between the values obtained for parallel titrations of a single suspension, does not necessarily indicate some fundamental difference in the viable cell populations derived from different tumours. Variation in the authenticity of the viable cell counts, in the batches of animals used for the titrations and in the ranges of inoculum size titrated, may account for variation of the TD50 from one experiment to another. Young, old, and even regressing tumours have been used indiscriminately for the preparation of single-cell suspensions, and this has not disclosed any association between the activity of the viable cells and the growth state of the tumour from which they were derived. White and Loeb (1910) have described the successful transplantation of regressing tumours, and Schrek (1936*a*) has found no effect of immunity on the growth capicity of the tumour cells. These findings do not suggest the presence of humoral factors in regressing tumours which are active against the tumour cells themselves and encourage the view that regression is the result of primary breakdown of the stroma.

The significantly lower TD50 values given by intramuscular sites of inoculation compared with subcutaneous sites, for S37, might ascribed to the greater vascularity of muscle. On the other hand, this site difference was not demonstrable for the C3H sarcoma, and a more likely explanation of the site difference found for S37 would appear to be that local resistance mechanisms are less readily manifested in the intramuscular sites.

From the results of the experiments recorded in Section 3, it is clear that a small inoculum of cells is not suppressed, in the period before it forms a palpable tumour, by multiple and larger inocula of the same tumour placed simultaneously elsewhere in the same animal. This being the case, it seems probable that the failure to give rise to palpable tumours which is displayed by half the inocula of about 2000 cells of S37, is not due to induction of systemic immunity by these inocula themselves during their prepalpable phase of growth. A further observation which leads to the same conclusion is that in numerous instances one of four inocula of about 2000 cells into a single animal may give rise to a tumour which grows progressively for weeks. If the failure of the other three inocula to give rise to tumours had been due to induced systemic immunity in the animal, the single tumour would not be expected to proceed as though no immunity were present. The fact that this same tumour may subsequently regress after reaching a quite considerable size suggests the operation of an induced resistance mechanism which takes some time to develop. These observations and considerations have led to the conception that the mechanism which brings about early extinction of the small inocula before they form palpable tumours is distinct from the induced mechanism which results in regression of a tumour that has attained moderate size.

The observation that " Hyalase " fails to influence the take of small single-cell inocula or to impose a distinctive form on the resulting tumours is contrary to what might be expected if some diffusible factor in the inoculum played any considerable part in the success of the implants.

Craigie (1952) suggested that dead cells or debris may interfere with the activity of viable tumour cells on transplantation. It is concluded from the results of the experiments described in Section 5, however, that dead tumour cells do not interfere with the proliferation of viable cells present in the same inoculum. A useful inference which may be made in this connection is that a large preponderance of dead cells in an inoculum should not interfere with the detection by inoculation of a small number of viable cells, these being the conditions met with when testing the survival of tumour tissue which has been subjected to a damaging procedure such as freezing.

As explained in Section 6, the observation that slightly lower TD50 values were obtained from titrations of suspensions of S37 ascitic cells as compared with suspensions prepared from solid tumours may be related to the relatively high proportion of dead (trypan blue stainable) cells which is characteristic of the latter suspensions. Since dead cells apparently do not interfere with the proliferation of viable sells (Section 5), and in view of the increase in the number of stained cells which was observed in suspensions prepared from solid tumours when they were allowed to stand, it is reasonable to conclude that the solid tumour preparations contain a relatively high proportion of non-viable cells which fail to stain with trypan blue. The viable cell counts of the solid tumour preparations therefore tend to be erroneously high. These considerations show that it is not permissible to conclude from the comparison that there is a difference between the activities of the viable cells from these two different sources.

The results of the titrations of the C3H sarcoma cells in C3H mice leave little doubt that single viable cells of this tumour are quite capable of giving rise to tumours on transplantation to the subcutaneous tissue. This finding emphasises the necessity for excluding absolutely the possibility of cell contamination in all experiments designed to provide evidence of non-cellular transmission of tumours. The ease with which tumours can be derived from inocula of very small numbers of cells should not be lost sight of when attempts are made to interpret the histological changes which occur in small implants of tumour tissue after transplantation. The necrosis of tumour implants which has been described by several authors to whom reference has already been made is probably of no significance to the question of non-cellular transmission of tumours. Indeed the evidence provided here shows that tumours which arise in the vicinity of implants of small fragments of tumour tissue can be derived exclusively from free single cells which contaminate the surface of the fragment. When single cubes of S37 of 1–2 mm. side were dissected from solid tumour and washed individually with Tyrode solution, an average of over 10,000 viable tumour cells could be recovered from the separate washings. The appearances of implants of Sarcoma 180 at various stages after transplantation, which have been described in considerable detail by Zahl and Drasher (1947) and which these authors had difficulty in interpreting, are consistent with the new tumours having arisen from the proliferation of individual cells that had been wiped off the surface of the implanted fragments on to the adjacent tissues of the host during implantation. Necrosis of the main mass of the implant could be accounted for by its failure to gain intimate contact with a source of oxygen and nutrients.

In Fig. 1 an attempt has been made to compare graphically the relationships between cell dose (expressed as the logarithm) and tumour frequency for the C3H sarcoma and for S37. The available mice of the strains used did not allow a more accurate definition of the points relating to the higher and lower tumour frequencies, and the figure serves to show only that the relationship between cell dose and tumour incidence is not greatly dissimilar in the two cases, at least in the region of the TD50. This suggests that the variability of responsiveness of the sites is not very different in the two cases. If this is so, an interpretation of the difference in the quantitative data relating to the transplantation of the two tumours centres round the differences in the TD50s. The multiple difference between these values from the graph is 27, but the figure will, of course, vary within certain limits in accordance with variations in the TD50 from one experiment to another.

On theoretical grounds it may be assumed that all of the C3H tumour cells are genetically compatible with their hosts, and that each is capable of giving rise to a rumour on transplantation. The comparison of the C3H sarcoma with S37 then suggests that, in the case of the latter tumour, only 1 out of 27 (3·7 per cent) of the viable cells are capable of giving rise to a tumour in the 4–6-week-old albino mice. But the incapacity of the majority of the cells to form new tumours on transplantation is not due to an intrinsic lack of proliferative power. Evidence reported elsewhere (Hewitt, 1953) shows that when S37 cells are titrated in new-born albino mice the TD50 levels obtained are no greater than that obtained for the C3H sarcoma titrated in adult C3H mice. The apparent failure of the majority of the S37 cells to survive transplantation is evidently attributable to the genetic difference between the tumour cells and their hosts, this difference being not manifest in immature mice.

It may be that the ratio 1 : 27 represents merely the average number of cells in the S37 inocula which survives the initial hazards of exposure to the natural resistance factors in the foreign host, and that those cells which survive do so fortuitously. The evidence from these studies, however, does not exclude the possibility that the cells in the inoculum which survive to form a tumour may owe their survival to an intrinsic peculiarity. The evidence of heterogeneity given by other workers, to which reference was made in the introduction, is not inconsistent with such a possibility.

The possibility that tumours of unknown genetic origin which are characterised by their capacity to grow across strain barriers, such as S37 may owe this faculty to the continuous production of a spectrum of cells of varying gene dependence is not discounted by genetic studies in which account has been taken only of the response to massive inocula. Barrett (1952) has described a change in the genetics of transplantation of a tumour following its growth in a strain foreign to the strain of origin. He considered that the change which occurred was not explained simply by loss of genetic specificity. It is certain that, at one stage during this change, the cells composing the tumour must have been heterogeneous in respect of the genetics of their transplantability.

The use of quantitative methods of transplantation in the study of what has recently been termed " immunogenetics " (Barrett, 1952) invites an experimental approach to this subject which appears to have received little attention. Such an approach would require the development of methods of single cell isolation analogous to those used in bacteriology, followed by comparison of the properties of tumours derived from such single cells, Mutational changes affecting the genetics of transplantation, growth rate and histological appearance are commonly observed in transplantable tumours (Snell, 1949). Segregation methods should greatly increase the scope of such observations.

### SUMMARY.

1. Methods are described of preparing single-cell suspensions of mouse sarcomata from solid and ascitic tumours, of finding the viable tumour cell density in the suspensions, and of " titrating " them for tumour producing activity on inoculation. From the results of the titrations a TD50 value (the number of viable sarcoma cells required to give tumours in half the inoculated sites) was calculated by a standard method.

2. Sixteen titrations of S37 performed in mice of impure stock, all members of which grew the tumour from massive inocula, gave a mean TD50 of 1641 with a coefficient of variation of 51 per cent.

3. The ratio of the TD50 values given by paired titrations of a S37 suspension ranged from 1·0 to 1·82 in 8 different experiments.

4. Intramuscular inocula of S37 cells gave a significantly lower TD50 than subcutaneous inocula.

5. The tumour-producing activity of cell doses near the TD50 did not appear to be suppressed by larger inocula placed simultaneously elsewhere in the same animal.

6. The TD50 was not modified by the addition of " Hyalase " to the inocula.

7. A large preponderance of dead cells and debris in the inocula did not reduce, but may have enhanced the production of tumours from viable cell doses near the TD50.

8. The TD50 values given by viable S37 cells obtained from solid tumours were not significantly different from those given by the corresponding cells of ascitic tumours.

9. The TD50 given by the viable tumour cells of a C3H sarcoma in C3H mice was about 25. Mice over one year old did not give a value higher than 3-month-old mice.

10. The significance is discussed of the notable difference in TD50 levels given by S37 in an impure strain of mouse and by the C3H sarcoma in the homologous mouse strain.

11. An appreciable incidence of tumours was obtained in C3H mice from cell doses of under 10 viable sarcoma cells. The implication of this finding for the problem of cell-free transfer is discussed.

I wish to express my gratitude to Miss Catherine Fysh, B.Sc., for skilled technical assistance, and to Professor R. J. V. Pulvertaft and Dr. R. J. O'Connor for valuable encouragement and advice. I am indebted also to the British Empire Cancer Campaign for grants during the tenure of which these investigations were made.

### REFERENCES.

ANDERVONT, H. B.—(1936) *Publ. Hlth. Rep., Wash.*, **51**, 591.
BARRETT, M. K.—(1952) *Cancer Res.*, **12**, 535.
BERENBLUM, I., AND SHUBIK, P.—(1949) *Brit. J. Cancer*, **3**, 384.
CRAIGIE, J.—(1952) *J. Path. Bact.*, **64**, 251.
*Idem*, LIND, P. E., HAYWARD, M. E., AND BEGG, A. M.—(1951) *Ibid.*, **63**, 177.
DE GAETANI, G. F., AND BLOTHNER, E.—(1936) *Z.-Krebsforsch.*, **44**, 108.
FURTH, J., AND KAHN, M. C.—(1937) *Amer. J. Cancer*, **31**, 276.
GYE, W. E., BEGG, A. M., MANN, I., AND CRAIGIE, J.—(1949) *Brit. J. Cancer*, **3**, 259.
HEWITT, H. B.—(1953) *Ibid.*, **7**, 384.
IRWIN, J. O., AND CHEESEMAN, E. A.—(1939) *J. Hyg., Camb.*, **39**, 574.
KAHN, M. C., AND FURTH, J.—(1938) *Proc. Soc. exp. Biol., N.Y.*, **38**, 485.
LASNITZKI, I.—(1952) *J. Path. Bact.*, **64**, 252.
PAPPENHEIMER, A. M.—(1917) *J. exp. Med.*, **25**, 633.
PASSEY, R. D., DMOCHOWSKY, L., LASNITZKI, J., AND MILLARD, A.—(1950) *Brit. med. J.*, ii, 1134.
REED, L. J., AND MUENCH, H.—(1938) *Amer. J. Hyg.*, **27**, 493.
ROUS, P., AND KIDD, J. G.—(1941) *J. exp. Med.*, **73**, 365.
SCHREK, R.—(1936*a*) *Amer. J. Cancer*, **28**, 345.—(1936*b*) *ibid.*, **28**, 389.
SNELL, G. D.—(1949) ' Proceedings of the National Cancer Conference,' p. 28. United States.
STRONG, L. C.—(1948) *Science*, **108**, 688.
WHITE, E. P. C., AND LOEB, L.—(1910) *Zbl. Bakt.*, **56**, 488.
ZAHL, P. A., AND DRASHER, M. L.—(1947) *Cancer Res.*, **7**, 658.